# Prevalence of malaria parasites in adults and its determinants in malaria endemic area of Kisumu County, Kenya

**DOI:** 10.1186/s12936-015-0781-5

**Published:** 2015-07-08

**Authors:** Rachel Jenkins, Raymond Omollo, Michael Ongecha, Peter Sifuna, Caleb Othieno, Linnet Ongeri, James Kingora, Bernhards Ogutu

**Affiliations:** Health Services Research Department, Institute of Psychiatry, Kings College London, de Crespigny Park, London, SE5 8AF UK; Kenya Medical Research Institute, Nairobi, Kenya; Kenya Medical Research Institute, Kisian, Kisumu, Kenya; Kombewa Health and Demographic Surveillance Site, Kombewa, Kenya; Department of Psychiatry, University of Nairobi, Nairobi, Kenya; Kenya Medical Training Kenya Medical Training College, Nairobi, Kenya

**Keywords:** Malaria, Parasitaemia, Prevalence, Risk factors, Gender, Household survey, Demographic surveillance site

## Abstract

**Background:**

The prevalence of malaria parasites in adults in Africa is less well researched than in children. Therefore, a demographic surveillance site was used to conduct a household survey of adults in the malaria endemic area of Maseno division in Kisumu County near Lake Victoria.

**Methods:**

A random survey of 1,190 adults living in a demographic health surveillance site in a malaria endemic area of 70,805 population size was conducted, measuring presence of malaria parasites by slide microscopy. Data were analysed using STATA to calculate the prevalence of malaria and associated risk factors.

**Results:**

The adult prevalence of presence of malaria parasites in Maseno was 28% (95% CI: 25.4–31.0%). Gender was a significant sociodemographic risk factor in both univariate (OR 1.5, p = 0.005) and multivariate (OR 1.4, p = 0.019) analyses. Females were 50% more likely to have malaria than men.

**Conclusions:**

Presence of malaria parasites is common in the adult population of this endemic area, and the rate is greatly increased in women. The presence of such an adult pool of malaria parasites represents a key reservoir factor in transmission of parasites to children, and is relevant for plans to eradicate malaria.

## Background

Globally, malaria causes around 627,000 deaths in 2013, mostly of children aged under 5 years living in Africa [1]. There are now a large number of regular prevalence surveys of childhood parasitaemia [1], as most malaria deaths occur in children, but published surveys of parasitaemia in the general adult population are very scarce [2]. Thus, of the 46 countries contributing household surveys of the presence of malaria parasites to the WHO 2013 report on malaria, all were of children [1]. However, the prevalence of parasitaemia in adults remains of scientific interest, not only because clinical attacks in adults remain an important cause of death in adults [3, 4], as well as of morbidity and health service use [5], but also because adults form a community reservoir of infection for children. With the current sustained implementation of malaria control and prevention strategies across most African countries and the consideration of elimination in some settings [6, 7], the impact of this adult reservoir in these control strategies needs to be assessed. In addition, as the epidemiology of malaria changes across Africa there are likely to be changes in the disease pattern with adults becoming susceptible to severe disease and this trend should be monitored.

In Kenya, there have been two national malaria indicator surveys recently, in 2007 and 2010. The adult population acquire partial immunity, the risk of death is higher in children and pregnant women, and malaria indicator surveys tend to focus on children [1]. The recent Kenya malaria indicator surveys of 2007 [8] and 2010 [9] have focussed on children aged 6 months to 5 years, and 6–14 years, respectively. Comparison of these surveys with previous malaria mapping exercises [10] of the presence of malaria parasites in children aged 2–10 years between 1975 and 2009 indicates that malaria is declining in prevalence across the country over the last 20 years, but endemic areas remain around Lake Victoria basin and in the Coast region [8], where transmission is high throughout the year.

The Kenya malaria survey 2007 [8] found a prevalence of 17% in children under 5 years in endemic areas, compared with 1.4% in areas of seasonal malaria transmission, 1% in epidemic prone areas, and 0.4% in low risk transmission areas (Kenya malaria indicator survey 2007). The Kenya malaria indicator survey, 2010, [9] covered children up to the age of 14 and found that the prevalence in children below 5 years increased from 4% in 2007 to 8% in 2010 while the prevalence in children aged 10–15 was 15% on average, ranging from 38% in the Lake endemic area to less than 5% elsewhere.

This paper reports the prevalence of the presence of malaria parasites in a random household sample of adults aged 18 and above in Maseno division of Kisumu county western Kenya in 2012/2013, and its relationship to key socio-demographic variables. It forms part of a wider survey of malaria, mental health and immunity, which will be published in a series of forthcoming papers.

## Methods

### Study population

The sample frame is a sub-county in Kenya, in an area endemic for malaria, namely Maseno division within Kisumu County, Western Kenya which has a population of 70,805 (Kombewa HDSS 2012). Females constitute 53% of the population. The mean household number is four people per household with a population density of about 374 people/km^2^. The population is largely young with a mean age of 23 years. Among the population sampled, those aged 0–14 years constitutes 46%, with those aged 15–64 years and 65+ years constituting 49 and 5%, respectively.

The population is primarily black African, and the languages spoken are Dholuo (predominant ethnic group), Kiswahili and English. The area is largely rural, with most residents living in villages, which are a loose conglomeration of family compounds near a garden plot and grazing land. The majority of the houses are mud-walled with either grass thatched or corrugated iron-sheet roofs. Water is sourced mainly from community wells, local streams and the lake for those living on the shores of Lake Victoria. Most water sources are not chlorinated. Subsistence farming, animal husbandry and fishing are the main economic activities in the area.

Malaria is holoendemic in this area, and transmission occurs throughout the year. The ‘long rainy season’ from late March to May produces intense transmission from April to August. The ‘short rainy season’ from October to December produces another, somewhat less intense, transmission season from November to January. This area was used for an earlier study on prevalence of common mental disorder and psychosis, and their risk factors in 2004 [11, 12].

### Study site

**Figure 1:**
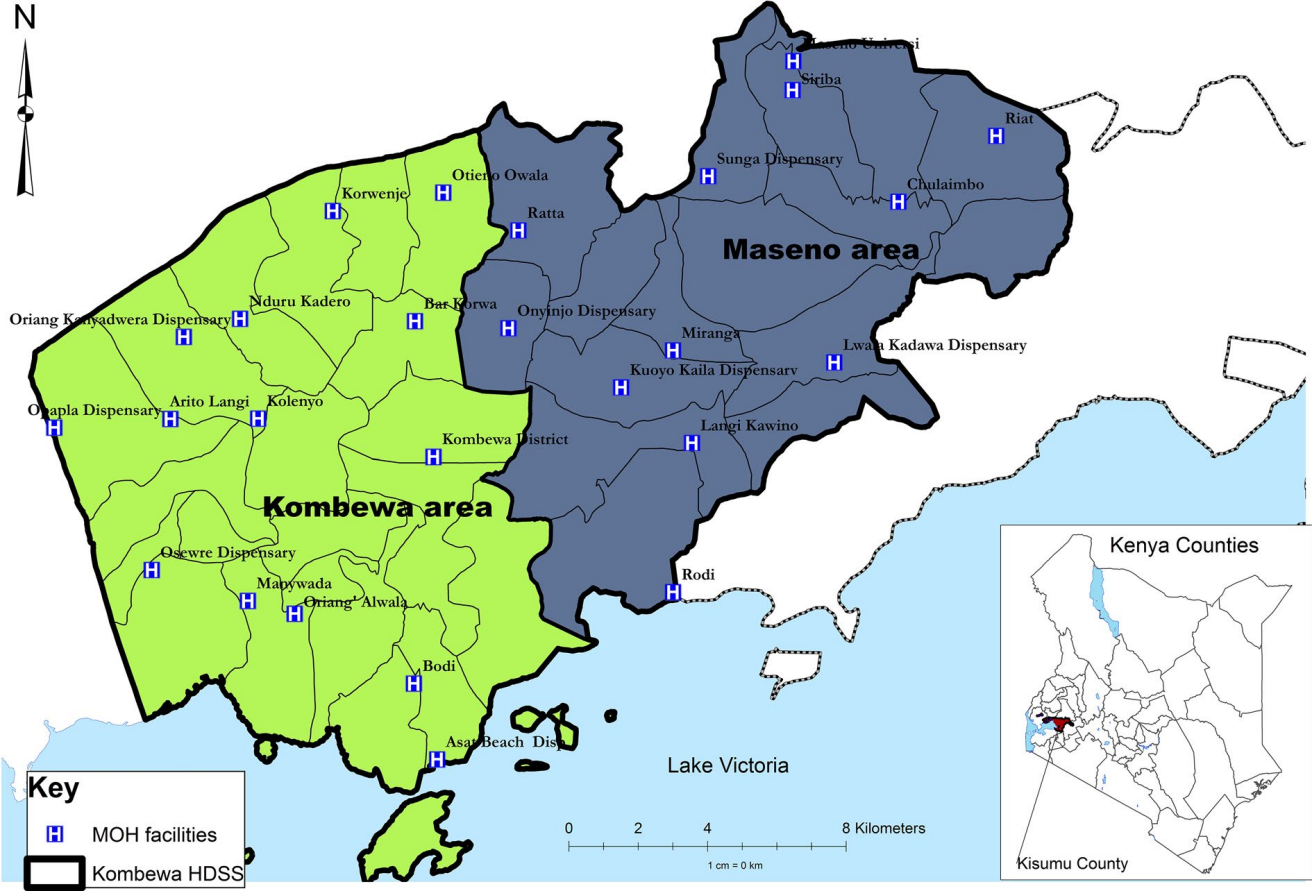
Map of study site.

Figure 1 shows the study site, Maseno area.

### Study participants

The study sample was selected from Maseno division which is sub-divided into four locations, 17 sub-locations and 184 enumeration areas (villages) based on mapping work done earlier by the Kombewa Health and Demographic Surveillance System (Kombewa HDSS) run by the KEMRI/Walter Reed Project. The Kombewa HDSS is a longitudinal population registration system set up to monitor the evolving health and demographic problems of the study population in Kombewa and Maseno divisions [13]. Some villages with less than 50 households were merged together to create new enumeration areas. A total sample size of 1,010 adults was needed for the survey and, since the sample frame included 170 enumeration areas, this necessitated the random selection of seven households in each enumeration area from which to select one adult to give a reasonable safety margin of a sample of 1,190 in order to allow for an estimated 15% refusal rate. Village maps were used to assign households and guide the research assistants during the survey. Using the Kish Grid Method [14], one individual was selected from each of the sampled household. The demographics and reasons for the refusal were recorded in notebooks by the research assistants. A total of 1,190 households were visited.

### Study procedures

Meetings were held with the community leaders to explain the purpose of the study and answer questions. The participants in the survey were approached in their own homes for voluntary written and witnessed informed consent, and then capillary blood samples were collected for preparation of thick and thin blood films (reported here) and immunity (which will be reported in accompanying papers). A structured epidemiological interview assessment accompanied the blood tests, which included detailed socio-demographic and housing variables (reported here) and additional sections on psychosocial variables (which will be reported in accompanying papers).

Field staff training was undertaken in November 2012 for 5 days in the KEMRI field centre in Kisumu. Team supervisors and research assistants were trained on the rationale and methodology of the interviews, use of personal digital assistants (PDAs) to collect the interview data, and the methodology for blood samples. Prior to the field work, the communities via their community leaders received information about the study and an opportunity to discuss the project. The field work commenced on December 2012, and continued intermittently until June 2013. The research assistants were driven to the study sites, and were closely supervised by field supervisors. The questionnaires were programmed on to the PDAs and tested before the actual field work. Socio-demographic data were captured on the PDAs. They were then transferred at the end of each day to a personal computer for merging, and transmission to the statistician for analysis.

### Sample collection and processing

Venipuncture blood samples of about 5 mL were collected aseptically into EDTA tubes from the enrolled participants before any treatment interventions. Blood smears were immediately prepared, dried and packed carefully in the field, and sent to KEMRI at Kisian, Kisumu, where staining and malaria parasite determination was performed by well trained laboratory technologists. Briefly, thick and thin peripheral blood smears were stained with Giemsa reagent and the prevalence of parasitaemia was determined by counting the asexual parasites against 300 leukocytes assuming a count of 8,000 leukocytes per microlitre of blood.

### Statistical analysis

The prevalence of malaria parasites was analysed in relation to a variety of socio-demographic, housing and economic variables. Predictors of parasitaemia were also examined using STATA [15] to calculate unadjusted and adjusted odds ratios. Households have been categorized into different socio-economic levels using an index of household assets, constructed applying the principal component analysis (PCA) procedure, as a proxy indicator for socio-economic status. In coming up with the asset quintiles, type of house, roofing and walling material, source of water, toilet facility and land have been used [16, 17].

### Ethics

Ethical approval was granted by the Kings College London and Kenya Medical Research Institute Ethics Review committees (PNM/11/12-54 and SSC 2374, respectively). Written informed consent was sought from participants to take part in the study, be interviewed and to give blood, and for the data to be analysed and published. Permission was obtained to conduct the study in households in Maseno area, which is part of the KEMRI/WRP Kombewa HDSS.

## Results

1,158 participants consented to the study while 32 refused to participate in the study interviews, and 149 refused to give a blood sample (the main reasons given were the fears that the sample would either be used for an HIV test or that it would be sold for profit), thus giving an overall response rate of 91.4%. The prevalence of malaria in the total adult population in this sample was 28.1%, and its association with sociodemographic factors is shown in Table 1.

**Table 1:** Relationship between malaria parasitaemia and various socio-demographic factors, using bivariate analysis (unadjusted odds ratios)

Factors	n	Prevalence	Unadjusted OR (95% CI)	p value
Gender				
Male	514	24.1	1	–
Female	452	32.3	1.5 (1.13–1.99)	0.005
Marital status				
Married/cohabiting	614	27.0	1	–
Single	154	27.9	1.0 (0.70–1.56)	0.825
Divorced/widowed	197	31.0	1.2 (0.85–1.72)	0.286
Education				
None	103	30.1	1	–
Primary	537	26.4	0.8 (0.53–1.33)	0.445
Secondary	265	29.4	1.0 (0.59–1.59)	0.900
Post secondary	61	31.2	1.1 (0.52–2.09)	0.888
Employment				
Unemployed	472	28.6	1	–
Self employed	415	28.2	1.0 (0.73–1.31)	0.893
Employed	79	22.8	0.7 (0.42–1.29)	0.287
Perceived economic situation				
Very easy/easy	103	27.2	1	–
Neither easy nor difficult	437	25.4	0.9 (0.56–1.48)	0.710
Very difficult/difficult	419	30.8	1.2 (0.74–1.93)	0.475
Perceived accommodation situation				
Very easy/easy	94	25.5	1	–
Neither easy nor difficult	447	26.6	1.1 (0.64 to 1.76)	0.828
Very difficult/difficult	418	29.9	1.2 (0.75 to 2.07)	0.400
Asset quintile				
Highest, Q1	338	26.3	1	–
Q2	329	29.8	1.2 (0.85 to 1.66)	0.321
1	335	28.4	1.1 (0.79 to 1.55)	0.555

*OR* odds ratio, *CI* confidence interval.

**Figure 2:**
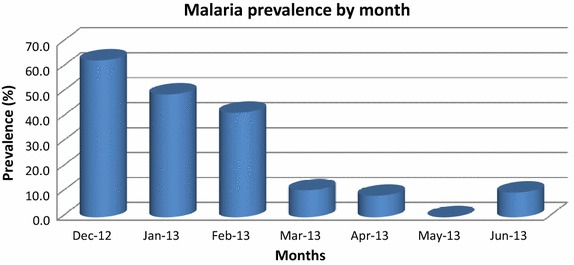
Seasonal variation in malaria prevalence.

There was no significant difference in the risk of malaria with age band, or on any other socio-demographic variable, with the exception of gender (see Table 1). Females had a 50% higher risk of having malaria compared to males (OR 1.5, p = 0.005) in the univariate analysis. There was however variation in malaria prevalence by month of interview, as the survey period ranged from December 2012 to June 2013. This is shown in Figure 2. Over the 6-month period prevalence rates ranging from around 40 to 60% in December, January and February to around 10% between March and June. In the adjusted analysis (Table 2), the risk of malaria is 40% higher in females compared to males (OR 1.4, p = 0.019).

**Table 2:** Relationship of malaria parasitaemia with gender, adjusted for the other sociodemographic variables using logistic regression analysis (adjusted odds ratios) risk factors for malaria:adjusted odds ratios

Factors	n	Adjusted OR^a^	95% CI	p value
Sex				
Male	604	1	–	–
Female	548	1.4	1.06–1.89	0.019

*OR* odds ratio, *CI* confidence interval.

^a^Variables identified as univariate predictors of malaria.

## Discussion

### Summary of findings

This study is the first published survey of the adult prevalence of malaria in Kenya. The survey is of a general household population living in a malaria endemic area, and found that the adult prevalence of malaria in Maseno division is 28% over a 6-month period, ranging from 40 to 60% in December, January and February to around 10% between March and June. We enquired in detail about aspects of housing and assets as well as socio-demographic factors, but the only significant socio-demographic risk factor that we were able to find in this sample size was gender in both univariate and multivariate analyses. Women were 40% more likely to have malaria than men (multivariate analysis). There are at least two plausible contributions to the higher prevalence in women. In the study area, the HIV prevalence is higher in women so this may contribute to the higher prevalence of malaria that we found in women, because HIV is known to cause loss of immunity and thus adults who have HIV are more likely to have malaria than those who do not have HIV [18, 19]. In addition, pregnant women specifically primigravidae and secondigravidae, lose the acquired semi-immunity of adulthood, and are more prone to malaria than other adults so this may also contribute to the higher prevalence found in women [20].

### Comparison of findings with other adult studies

The finding of an adult prevalence of malaria of 28% in an area endemic for malaria is double the 14% microscopy prevalence found in a recent household survey of a malaria endemic area in Mozambique [2]. A 22-year longitudinal study in a village in Senegal found that parasite prevalence declined in adults from 58% in 1990 to 0.3% in 2012 following systematic eradication and treatment interventions. [21].

Previous studies have found that in malaria endemic zones, the risks of malaria attacks declines with age between childhood and adulthood, indicating that protective immunity is acquired with increasing age up to adulthood [22]. This study did not find a change of parasite prevalence across the adult age range, suggesting that there is no further increase in protective immunity, with increasing adult age. Previous studies on general population prevalence of malaria have not been analysed by gender. Mayor et al. [2] did not disaggregate their data on adult malaria by gender, and the national Kenya malaria survey 2010 does not disaggregate childhood parasite prevalence by gender [9]. It is of interest that the malaria prevalence was marginally lower in adults in the highest wealth quartile, similar to the findings of the Kenya malaria survey 2010 in children.

### Implications of survey

Despite earlier vigorous calls for adult malaria surveys [4] in order to better monitor the transmission of malaria, adult household surveys of malaria are scarce, and it has been strongly argued that epidemiological studies of malaria in Africa are essential for eradication strategies [7]. This study has demonstrated the relatively high prevalence of malaria parasites in adults in an endemic area, ranging from 60% at the height of the malaria season to around 10% in the other months. The mortality of children from malaria remains high in endemic areas, and so there is considerable policy and practice attention to factors reducing transmission, such as use of bed nets, indoor residual spraying, testing of all persons with fever, and treating confirmed cases with artemether–lumefantrine. Despite these efforts, such a high adult prevalence of parasites emphasises the relevance of the adult malaria parasite reservoir on the transmission of disease in both children and adults [23] and it supports the need for further consideration and research evaluation of treatment of asymptomatics, including approaches such as intermittent screening and treatment (IST) as part of an integrated malaria control programme, if malaria elimination is to be achieved [24].

This study represents a contribution to the epidemiology of malaria in adults in an endemic area of Kenya. The probability sample of households and adults was drawn from a demographic surveillance site.

The field work, which had been planned to last 3 months, had to be extended to 6 months to cover interruptions during the 2013 national election, due to concerns about potential election violence. Participating women were not asked if they were pregnant, and this is likely to be a major factor in the increased prevalence of malaria in women compared to men. We did not examine parasite prevalence by polymerase chain reaction (PCR), which generally finds a higher prevalence than microscopy alone.

## Conclusions

The prevalence of malaria parasites in adults in an area endemic for malaria in Kenya remains relatively high at 28%. The presence of such an adult pool of parasites represents a key factor in transmission of parasites to children, and is relevant to the malaria eradication agenda.
